# Serum creatinine/cystatin C ratio is a surrogate marker for sarcopenia in patients with idiopathic pulmonary fibrosis

**DOI:** 10.1186/s12890-022-02000-3

**Published:** 2022-05-23

**Authors:** Kohei Fujita, Hirotsugu Ohkubo, Akiko Nakano, Norihisa Takeda, Kensuke Fukumitsu, Satoshi Fukuda, Yoshihiro Kanemitsu, Takehiro Uemura, Tomoko Tajiri, Ken Maeno, Yutaka Ito, Tetsuya Oguri, Yoshiyuki Ozawa, Takayuki Murase, Akio Niimi

**Affiliations:** 1 Department of Respiratory Medicine, Allergy and Clinical Immunology, Nagoya City University Graduate School of Medical Sciences, 1 Kawasumi, Mizuho-cho, Mizuho-ku, Nagoya, Aichi 467-8601 Japan; 2 Department of Respiratory Medicine, Nagoya City University East Medical Center, Nagoya, Japan; 3 Department of Radiology, Nagoya City University Graduate School of Medical Sciences, Nagoya, Japan; 4 Department of Pathology and Molecular Diagnostics, Graduate School of Medical Sciences, Nagoya City University, Nagoya, Japan

**Keywords:** Idiopathic pulmonary fibrosis, Sarcopenia, Cystatin C, Creatinine, Bioelectrical impedance, Patient-reported outcomes

## Abstract

**Background:**

The serum creatinine/cystatin C (Cr/CysC) ratio has attracted attention as a marker for sarcopenia, but has not been studied in patients with idiopathic pulmonary fibrosis (IPF). This study aimed to confirm the utility of the serum Cr/CysC ratio in predicting sarcopenia and investigate its clinical relevance.

**Methods:**

This cross-sectional pilot study prospectively enrolled patients with stable IPF. IPF was diagnosed through multidisciplinary discussions according to the 2018 international guidelines, and sarcopenia was diagnosed according to the 2019 consensus report of the Asian Working Group for Sarcopenia. Patient-reported outcomes (PROs) were evaluated using the modified Medical Research Council (mMRC) dyspnea scale, chronic obstructive pulmonary disease assessment test (CAT), and King’s Brief Interstitial Lung Disease (K-BILD) questionnaire. The associations between serum Cr/CysC ratio and the presence of sarcopenia and other clinical parameters, including PROs scores, were examined.

**Results:**

The study enrolled 49 Japanese patients with IPF with a mean age of 73.0 ± 7.7 years and a mean percentage of predicted forced vital capacity of 80.4 ± 15.5%. Sarcopenia was diagnosed in 18 patients (36.7%), and the serum Cr/CysC ratio was 0.86 [0.76–0.94] (median [interquartile range]). The receiver operating characteristic curve analyses for the detection of sarcopenia according to the serum Cr/CysC showed that the area under the curve, optimal cutoff value, specificity, and sensitivity were 0.85, 0.88, 0.65, and 0.94, respectively. Sarcopenia was identified in 13% of patients with a high serum Cr/CysC ratio (≥ 0.88) and 60% of patients with a low serum Cr/CysC ratio (< 0.88) (*P* < 0.001). Multiple linear regression analysis showed that the serum Cr/CysC ratio was an independent predictive marker of worse PROs evaluated using mMRC (*P* < 0.05), CAT (*P* < 0.05), and K-BILD (*P* < 0.05).

**Conclusions:**

This study showed that the serum Cr/CysC ratio may be a surrogate marker of sarcopenia in patients with IPF. Furthermore, it is important to pay attention to the serum Cr/CysC ratio because a lower serum Cr/CysC ratio is associated with worse PROs. Further studies are required to validate these observations to determine whether the Cr/CysC ratio can be used to detect sarcopenia in patients with IPF.

## Background

Idiopathic pulmonary fibrosis (IPF) is a fibrotic and progressive pulmonary disease with a poor prognosis [1, 2]. As the disease progresses, patients with IPF experience exercise intolerance, physical inactivity, and impaired health-related quality of life. A prospective cohort of patients with interstitial lung diseases, including 40 patients with IPF, showed significantly lower muscle mass in patients with impaired pulmonary function [3]. Patient-reported outcomes (PROs) measures, such as questionnaires or surveys that ask patients about their perceptions of issues, such as symptoms or health-related quality of life, play an important role in the symptomatic multifaceted management of patients with IPF [4].

Sarcopenia is an age-related syndrome characterized by a progressive and generalized loss of skeletal muscle mass and function and is strictly correlated with physical disability, poor quality of life, and death [5, 6]. According to the consensus report of the Asian Working Group for Sarcopenia (AWGS) 2019 [6], sarcopenia is diagnosed based on the loss of muscle mass, low muscle strength, and/or low physical performance. For muscle assessments in IPF patients, the use of a fat-free mass index [7] and the cross-sectional area of the erector spinae muscles [8–10] have been reported. However, sarcopenia defined using appendicular skeletal mass index (ASMI) in a cohort of patients with IPF has not been fully evaluated.

Serum creatinine and serum cystatin C levels are usually used to assess renal function [11–16]. However, because serum creatinine levels are affected by muscle mass, they are decreased in patients with sarcopenia. In contrast, serum cystatin C levels are not affected by muscle mass [17–19]. Based on these characteristics, the serum creatinine/cystatin C (Cr/CysC) ratio can predict muscle mass; thus, this ratio has been studied in many diseases [11–16]. However, to the best of our knowledge, no study has examined the utility of the serum Cr/CysC ratio in patients with IPF.

We hypothesized that the serum Cr/CysC ratio may be a surrogate marker for detecting sarcopenia in patients with IPF. In this cross-sectional study, we diagnosed sarcopenia according to AWGS 2019 [6] and examined the association between the serum Cr/CysC ratio and the presence of sarcopenia and other clinical parameters.

## Methods

### Ethics

This study was conducted in accordance with the amended Declaration of Helsinki, and was approved by the ethics review board of the Nagoya City University Hospital (approval number 60-20-0190). Written informed consent was obtained from all participants.

### Patients

IPF was diagnosed through multidisciplinary discussions according to the 2018 international guidelines [1]. The following inclusion criteria were applied: written informed consent obtained for this study; and the ability to perform a 6-min walk test (6MWT). The following exclusion criteria were applied: long-term oxygen treatment at rest; active cancer; and inability to understand the questionnaires regarding PROs. Between April 2021 and December 2021, outpatients with stable IPF were screened at the Nagoya City University Hospital (Nagoya, Japan), and 60 patients were identified. Four patients received long-term oxygen therapy at rest. One patient was unable to undergo 6MWT because of unstable angina. Two patients were unable to perform 6MWT because they typically used wheelchairs. Four patients declined to participate in the study. Thus, 49 outpatients were enrolled in the study.

### Diagnosis of sarcopenia

Sarcopenia was diagnosed according to the current criteria of AWGS 2019 [6]. Sarcopenia was diagnosed based on the loss of muscle mass, low muscle strength, and/or low physical performance. The detailed methods and criteria are described below. Muscle mass: The ASMI was calculated using a multifrequency bioelectrical impedance (BIA) analyzer (InBody 720; InBody Japan, Tokyo, Japan). The cutoff values for low muscle mass were < 7.0 kg/m^2^ for males and < 5.7 kg/m^2^ for females. Muscle strength: Handgrip strength was measured in the standing position with full elbow extension using an electronic dynamometer (HG-251; N-Force, Wakayama, Japan). The measurements were obtained twice for each hand, with the largest value used as the grip strength value for analysis. The cutoff values for low muscle strength were defined as < 28.0 kg for men and < 18.0 kg for women. Physical performance: Usual gait speed was used to evaluate physical performance. It was calculated by measuring the time taken by the study participants to walk a 10-m corridor. The participants were instructed to walk down the corridor at their usual speed. The cutoff value for low physical performance was defined as < 1.0 m/s for both men and women.

### Pulmonary function tests and 6-min walk test

All patients completed the pulmonary function tests using spirometry (CHESTAC-8900; Chest, Tokyo, Japan) according to the American Thoracic Society/Respiratory Society (ATS/ERS) criteria [20]. The diffusion capacity of carbon monoxide (DLco) was also measured (CHESTAC-8900). The percentage of predicted FVC (%FVC), percentage of predicted forced expiratory volume in 1.0 secomd (FEV_1_), and percentage of predicted DLco (%DLco) were calculated based on the patient’s height, age, and sex, according to Japanese standardized methods [21]. The 6MWT was performed without supplemental oxygen, in accordance with ATS guidelines [22].

### Clinical staging of IPF

Clinical staging of IPF was conducted according to the gender, age, and physiology (GAP) staging system [23].

### Strength, assistance in walking, rising from a chair, climbing stairs, and falls (SARC-F) questionnaire

The SARC-F Questionnaire was used to measure probable sarcopenia. This questionnaire consists of five items: strength, assistance in walking, rising from a chair, climbing stairs, and falls [24]. The SARC-F scores range from 0 to 10, with 0 to 2 points for each component.

### Patient reported outcomes

PROs are based on assessments of breathlessness, symptoms, and health-related quality of life. Breathlessness was evaluated using the 5-grade modified Medical Research Council dyspnea scale (mMRC). The symptoms and health-related quality of life were evaluated using the chronic obstructive pulmonary disease assessment test (CAT) [25, 26] and King’s Brief Interstitial Lung Disease (K-BILD) questionnaire [27, 28]. CAT is a self-administered quality of life questionnaire that measures the impact of lung disease on health status. It consists of eight items, each of which is scored on a scale of one to five. The total score ranges from 0 to 40. Higher scores indicate a greater impact on patients’ lives. K-BILD comprises 15 items in three domains: psychological health, breathlessness and activities, and chest symptoms. The K-BILD score ranges from 0 to 100, with higher values indicating better health.

### Statistical analyses

Continuous variables were tested for normality using the Shapiro–Wilk test and are presented as mean ± standard deviation, whereas non-normally distributed variables are presented as median and interquartile range. The relationships between the serum Cr/CysC ratio and other continuous variables were evaluated using Spearman’s rank correlation coefficients. The differences among groups were analyzed using the Student’s *t* test or the Mann–Whitney U test, as appropriate. The chi-squared test or Fisher's exact test was used to assess the differences in the percentages of patients. Receiver operating characteristic (ROC) curve analyses were performed to predict sarcopenia according to the serum Cr/CysC ratio, serum creatinine, and serum cystatin C level. Multiple linear regression analysis was performed to confirm that the serum Cr/CysC ratio was a predictive marker of patient-reported outcomes. To assess multicollinearity, the variance inflation factor (VIF) values were computed. Statistical significance was set at P < 0.05. Statistical analyses were performed using IBM SPSS Statistics version 28 (IBM Corp., Armonk, NY, USA).

## Results

### Patient characteristics

Patient characteristics are presented in Table 1. A total of 49 Japanese patients with IPF (mean age, 73.0 ± 7.7 years, mean %FVC, 80.4 ± 15.5%) were enrolled in this study. Sarcopenia was diagnosed in 18 patients (36.7%), and the serum Cr/CysC ratio was 0.86 [0.76–0.94].

**Table 1:** Characteristics of patients included in the study

	IPF (n = 49)
Age, years	73.0 ± 7.7
Sex, female, n (%)	5 (10.2%)
Body mass index, kg/m.^2^	22.3 ± 3.11
Smoking history	
Current or ex-smoker, n (%)	41 (83.7%)
Pack-years	30 [10–46]
Histological diagnosis, n (%)	15 (30.6%)
Severity	
GAP index	3 [3, 4]
GAP stage (I/II/III), n (%)	30 (61%)/19 (39%)/0 (0%)
Pulmonary function	
FVC, % predicted	80.4 ± 15.5
FEV_1_/FVC, %	81.9 ± 8.81
DL_CO_, % predicted	67.4 ± 19.1
PaO_2_, mmHg	87.9 [79.1–98.1]
Physical assessment	
Handgrip strength, kg	
Male	7.0 ± 0.9
Female	5.3 ± 0.5
Usual gait speed, m/s	1.10 ± 0.26
6MWT	
Distance, m	416 ± 88
Lowest SpO_2_, %	89 [85–92]
ASMI, kg/m^2^	
Male	7.0 ± 0.9
Female	5.3 ± 0.5
SARC-F	2 [1–3.5]
Patient reported outcome	
mMRC	1 [0–2]
CAT	13 [6.5–21]
K-BILD	
Psychological	56.9 [48.4–64.3]
Breathlessness and activities	50.2 [41.9–58.5]
Chest symptoms	73.4 [63.7–79.3]
Total	59.5 [54.2–62.7]
Blood examination	
Creatinine (mg/dL)	0.84 [0.75–0.99]
Cystatin C (mg/L)	1.00 [0.89–1.21]
Cr/CysC ratio	0.86 [0.76–0.94]
Comorbidity	
Hypertension, n (%)	28 (57.1%)
Dyslipidemia, n (%)	18 (36.7%)
Diabetes mellitus, n (%)	13 (26.5%)
Coronary artery disease, n (%)	5 (10.2%)
Chronic kidney disease, n (%)	2 (4.1%)
Sarcopenia, n (%)	18 (36.7%)

Data are presented as the mean (± standard deviation), median [interquartile range], or number (%)

*GAP* gender, age, and physiology, *FVC* forced vital capacity, *FEV*_*1*_ forced expiratory volume in 1.0 s, *DL*_*CO*_ diffuse capacity of the lung for carbon monoxide, *PaO*_*2*_ partial pressure for oxygen, *6MWT* 6-min walk test, *SpO*_*2*_ oxygen saturation by pulse oximetry, *ASMI* appendicular skeletal muscle index, *SARC-F* strength, assistance in walking, rising from a chair, climbing stairs, and falls, *mMRC* modified Medical Research Council, *CAT* chronic obstructive pulmonary disease assessment test, *K-BILD* King’s Brief Interstitial Lung Disease, *Cr/CysC ratio* creatinine/cystatin C ratio

### Correlations between serum creatinine/cystatin C ratio and appendicular skeletal muscle index, handgrip strength, and usual gait speed

**Fig. 1:**
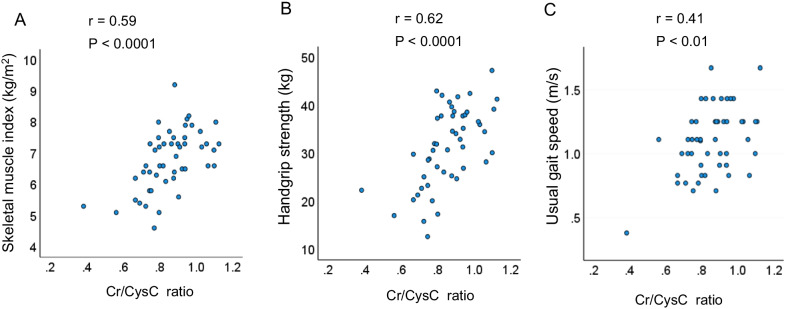
The correlations of serum Cr/CysC ratio with ASMI, handgrip strength, and usual gait speed. The serum creatinine/cystatin C (Cr/CysC) ratio was significantly correlated with **A** appendicular skeletal muscle index, **B** handgrip strength, and **C** usual gait speed

The correlations between the serum Cr/CysC ratio and ASMI, handgrip strength, and usual gait speed are shown in Fig. 1. The serum Cr/CysC ratio was significantly correlated with ASMI (r = 0.59,* P* < 0.001), handgrip strength (r = 0.62, *P* < 0.001), and usual gait speed (r = 0.41, *P* < 0.01).

### Relationships between serum creatinine/cystatin C ratio and clinical parameters

As presented in Table 2, the serum Cr/CysC ratio was correlated with age (r = 0.56, *P* < 0.001), body mass index (r = 0.42, *P* < 0.01), GAP index (r = -0.41, *P* < 0.01), PaO_2_ (r = 0.54, *P* < 0.001), distance walked during 6MWT (r = 0.42, *P* < 0.01), lowest SpO_2_ during 6MWT (r = 0.31, *P* < 0.05), ASMI (r = 0.59, *P* < 0.001), SARC-F (r = − 4.82, *P* < 0.001), mMRC (r = − 0.54, *P* < 0.001), CAT (r = − 0.38, *P* < 0.01), K-BILD-breathlessness and activity score (r = 0.52, *P* < 0.001), and K-BILD total score (r = 0.40, *P* < 0.005). In contrast, the serum Cr/CysC ratio was not significantly correlated with %FVC (r = 0.26, *P* = 0.07) or %DLco (r = 0.18, *P* = 0.21).

**Table 2:** Relationships between serum creatinine/cystatin C ratio and clinical parameters

	r	95% CI	*P* value
Age, years	− 0.56	− 7.31 to − 3.25	< 0.001
Body mass index, kg/m^2^	0.42	0.16–0.64	< 0.01
GAP index	− 0.41	− 0.62 to 0.14	< 0.01
FVC, % predicted, %	0.26	− 0.28 to 0.51	0.07
DL_CO_, % predicted, %	0.18	− 1.1 to 0.45	0.21
PaO_2_, mmHg	0.54	0.19–0.66	< 0.001
6MWT			
Distance, m	0.42	0.15–0.63	< 0.01
Lowest SpO_2_, %	0.31	0.026–0.55	< 0.05
SARC-F	− 4.82	− 6.76 to − 2.24	< 0.001
mMRC	− 0.54	− 0.72 to − 0.30	< 0.001
CAT	− 0.38	− 0.61 to − 0.99	< 0.01
K-BILD			
Psychological	0.27	− 0.20 to 0.52	0.06
Breathlessness and activities	0.52	0.27–0.71	< 0.001
Chest symptoms	0.25	− 0.05 to 0.50	0.09
Total	0.4	0.13–0.61	< 0.005

*GAP* gender, age, and physiology, *FVC* forced vital capacity, *DL*_*CO*_ diffuse capacity of the lung for carbon monoxide, *PaO*_*2*_ partial pressure of oxygen, *6 MWT* 6-min walk test, *SpO*_*2*_ oxygen saturation by pulse oximetry, *ASMI* appendicular skeletal muscle index, *SARC-F* strength, assistance in walking, rising from a chair, climbing stairs, and falls, *mMRC* modified Medical Research Council, *CAT* chronic obstructive pulmonary disease assessment test, *K-BILD* King's brief interstitial lung disease

### Receiver operating characteristic (ROC) curve analyses for detecting sarcopenia

**Fig. 2:**
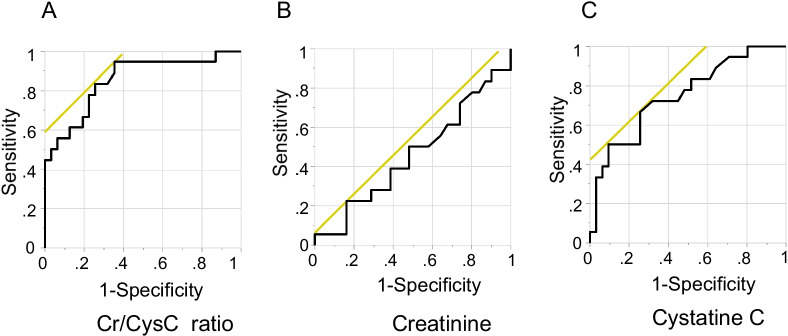
Receiver operator characteristic curves presenting sarcopenia. Receiver operator characteristic curves presenting sarcopenia according to **A** serum creatinine/cystatin C (Cr/CysC) ratio, **B** serum creatinine, and **C** serum cystatin C. The areas under the curve were 0.85, 0.46, and 0.75, respectively. As for Cr/CysC ratio, optimal cutoff value of 0.88 (specificity: 0.65, sensitivity: 0.94) was identified

ROC curve analyses of sarcopenia according to the serum Cr/CysC ratio, creatinine level, and cystatin C level are shown in Fig. 2. The areas under the curve (AUC) by ROC curve analyses of the serum Cr/CysC ratio, creatinine, and cystatin C were 0.85 (95% confidence interval [CI], 0.73–0.97, *P* < 0.001), 0.46 (95% CI, 0.28–0.63, *P* = 0.60), and 0.75 (95% CI, 0.60–0.89, *P* < 0.01), respectively. The AUC by ROC analysis of the serum Cr/CysC ratio was significantly higher than that of cystatin C (*P* < 0.001). We calculated the serum Cr/CysC cutoff to be 0.88, using the Youden index that maximizes the value of “sensitivity + specificity-1”. Based on the calculated cutoff value of 0.88, the specificity was 0.65, and the sensitivity was 0.94. The secondly candidate cutoff value had been 0.83 (specificity, 0.74; sensitivity, 0.83). Since the cutoff value of 0.88 has high sensitivity, it may be considered appropriate for the screening of sarcopenia in patients with IPF.

In addition, we performed ROC curve analysis of SARC-F score predicting sarcopenia. The AUC of the SARC-F score was 0.75 (95% CI, 0.60–0.91, *P* < 0.005).

### Clinical characteristics of the high-versus the low-serum creatinine/cystatin C ratio group

Sarcopenia was detected in 13% (n = 3) of the patients with a high serum Cr/CysC ratio (≥ 0.88) and in 60% (n = 15) of patients with a low serum Cr/CysC ratio (< 0.88) (chi-squared test, *P* < 0.001). Patient characteristics in patients with a high serum Cr/CysC ratio (≥ 0.88) and patients with a low serum Cr/CysC ratio (< 0.88) are shown in Table 3. There were significant differences in age (*P* < 0.001), body mass index (*P* < 0.01), GAP stage (*P* < 0.001), PaO_2_ (*P* < 0.005), handgrip strength (*P* < 0.001), ASMI (*P* < 0.001), SARC-F (*P* < 0.05), mMRC (*P* < 0.001), CAT (*P* < 0.05), K-BILD-breathlessness and activity score (*P* < 0.01), and serum cystatin C (*P* < 0.01). There were no significant differences in %FVC (*P* = 0.485) or %DLco (*P* = 0.651).

**Table 3:** Clinical characteristics of the high-versus the low-serum creatinine/cystatin C ratio group

	High (Cr/CysC ≧ 0.88)	Low (Cr/CysC < 0.88)	*P* value
Number of patients	24	25	
Age, years	69.0 ± 8.3	76.8 ± 4.8	< 0.001
Sex, female, n (%)	1 (4.2)	4 (16.0)	0.35
Body mass index, kg/m^2^	23.5 ± 2.3	21.1 ± 3.4	< 0.01
Severity			
GAP index	3 [2.5–4]	3 [3, 4]	0.06
GAP stage (I/II/III), n (%)	17/ (71)/7(29)/0(0)	13(52)/12(48)/0(0)	< 0.001
Pulmonary function			
FVC, % predicted	82.0 ± 13.5	78.9 ± 17.4	0.49
FEV_1,_ % predicted	81.2 ± 13.0	82.6 ± 16.5	0.74
DL_CO_, % predicted	68.7 ± 15.5	66.3 ± 22.2	0.65
PaO_2_, mmHg	92.3 [87.1–101]	82.0 [74.4–88.7]	< 0.005
Physical assessment			
Handgrip strength, kg	35.5 ± 5.7	27.4 ± 8.4	< 0.001
Usual gait speed, m/s	1.17 ± 0.23	1.04 ± 0.28	0.08
6MWT			
Distance, m	441 ± 81	392 ± 90	0.05
Lowest SpO_2_, %	90 [86–93]	88 [82–92]	0.13
ASMI, kg/m^2^	7.25 ± 0.78	6.34 ± 0.93	< 0.001
SARC-F	1.5 [0–2]	3 [1–5]	< 0.05
Patient-reported outcomes			
mMRC	0 [0–1]	2 [1–3]	< 0.001
CAT	10 [5–17]	13 [8.5–25]	< 0.05
K-BILD			
Psychological	57 [48–64]	55 [42–63]	0.22
Breathlessness and activities	50 [42–56]	46 [32–54]	< 0.01
Chest symptoms	73 [64–79]	73 [54–85]	0.20
Total	60 [54–63]	55 [47–61]	0.05
Blood examination			
Creatinine (mg/dL)	0.90 [0.79–1.08]	0.77 [0.72–0.97]	0.06
Cystatin C (mg/L)	0.91 [0.82–1.13]	1.08 [0.95–1.26]	< 0.01
Cr/CysC ratio	0.94 [0.90–1.05]	0.77 [0.71–0.80]	< 0.001
Comorbidity			
Hypertension, n (%)	14 (58%)	14(56%)	0.87
Dyslipidemia, n (%)	10 (42%)	8 (20%)	0.48
Diabetes mellitus, n (%)	7 (29%)	6 (24%)	0.68
Coronary artery disease, n (%)	4 (17%)	1(0.4%)	0.14
Chronic kidney disease, n (%)	2 (0.8%)	0 (0%)	0.14
Sarcopenia, n (%)	3 (13%)	15 (60%)	< 0.001

Data are presented as the mean (± standard deviation), median [interquartile range], or number (%)

*Cr/CysC ratio* creatinine/cystatin C ratio, *GAP* gender, age, and physiology, *FVC* forced vital capacity, *FEV*_*1*_ forced expiratory volume in 1.0 s, *DL*_*CO*_ diffuse capacity of the lung for carbon monoxide, *PaO*_*2*_ partial pressure for oxygen, *6MWT* 6-min walk test, *SpO*_*2*_ oxygen saturation by pulse oximetry, *ASMI* appendicular skeletal muscle index, *SARC-F* strength, assistance in walking, rising from a chair, climbing stairs, and falls, *mMRC* modified Medical Research Council, *CAT* chronic obstructive pulmonary disease assessment test, *K-BILD* King's Brief Interstitial Lung Disease

### Multiple linear regression analysis for patient-reported outcomes scores

The relationships between the serum Cr/CysC ratio and PROs scores were good, and significant differences in PROs scores were observed between the high and low serum Cr/CysC groups. The results of the multiple linear regression analyses of PROs scores are shown in Table 4. The serum Cr/CysC ratio was an independent factor contributing to the mMRC score (*P* < 0.05), CAT (*P* < 0.05), K-BILD-breathlessness score (*P* < 0.01), and K-BILD-total score (*P* < 0.05). In contrast to the serum Cr/CysC ratio, pulmonary function parameters such as %FVC and %DLco were not independent predictive factors. The VIF values of the serum Cr/CysC ratio, age, %FVC, %DLco, distance walked during 6MWT, and lowest SpO_2_ during 6MWT were 1.1918, 1.932, 1.973, 1.842, 1.922, and 1.770, respectively. All VIF values were < 10, and there was no multicollinearity among these factors.

**Table 4:** Multiple linear regression analysis for the scores of patient-reported outcomes (PROs)

	B	95% CI	β	*P* value
mMRC				
Serum Cr/CysC ratio	− 2.54	− 4.64 to − 0.44	− 0.23	< 0.05
Age	− 0.26	− 0.66 to 0.01	− 0.17	0.19
FVC, percent predicted	0.02	− 0.01 to − 0.39	0.25	0.06
DL_CO_, percent predicted	− 0.01	− 0.02 to 0.01	− 0.08	0.49
6MWT, distance	− 0.01	− 0.10 to − 0.03	− 0.44	< 0.001
6MWT, lowest SpO_2_	− 0.11	− 0.16 to 0.07	− 0.58	< 0.001
CAT				
Serum Cr/CysC ratio	− 23.5	− 43.1 to − 3.72	− 0.34	< 0.05
Age	− 0.32	− 0.69 to 0.06	− 0.28	0.10
FVC, percent predicted	0.13	− 0.06 to 0.32	0.22	0.18
DL_CO_, percent predicted	0.01	− 0.14 to 0.16	0.02	0.93
6MWT, distance	− 0.02	− 0.05 to 0.02	− 0.17	0.30
6MWT, lowest SpO_2_	− 0.82	− 1.26 to − 0.38	− 0.59	< 0.01
K-BILD (breathlessness and activities)				
Serum Cr/CysC ratio	66.9	29.5–104.3	0.53	< 0.01
Age	0.92	0.21–1.63	0.39	< 0.05
FVC, percent predicted	− 0.32	− 0.67 to 0.04	0.27	0.08
DL_CO_, percent predicted	− 0.09	− 0.19 to 0.37	0.09	0.53
6MWT, distance	0.06	− 0.01 to 0.12	0.29	0.06
6MWT, lowest SpO_2_	1.35	0.51–2.18	0.46	< 0.01
K-BILD (total)				
Serum Cr/CysC ratio	30.4	2.31–58.5	0.38	< 0.05
Age	0.57	0.04–1.10	0.38	< 0.05
FVC, percent predicted	− 0.17	− 0.44 to 0.10	− 0.23	0.21
DL_CO_, percent predicted	0.04	− 0.17 to 0.26	0.07	0.67
6MWT, distance	0.04	− 0.01 to 0.08	0.28	0.13
6MWT, lowest SpO_2_	0.70	0.07–1.33	0.38	< 0.05

*mMRC* modified Medical Research Council, *Serum Cr/CysC ratio* serum creatinine/cystatin C ratio, *FVC* forced vital capacity, *DL*_*CO*_ diffuse capacity of the lung for carbon monoxide, *6MWT* 6-min walk test, *SpO*_*2*_ oxygen saturation by pulse oximetry, *CAT* chronic obstructive pulmonary disease assessment test, *K-BILD* King's brief interstitial lung disease

## Discussion

The present study showed that the serum Cr/CysC ratio may be a good predictor of sarcopenia based on the consensus report of AWGS 2019 [6]. We calculated the best serum Cr/CysC cutoff value to be 0.88 (specificity, 0.65; sensitivity, 0.94). Based on this cutoff value of 0.88, the percentage of sarcopenia was 60% in the low serum Cr/CysC ratio group and 13% in the high serum Cr/CysC ratio group in the present study. Thus, the serum Cr/CysC ratio may be a useful marker for screening sarcopenia.

According to the consensus report of AWGS 2019 [6], measurements of ASMI, handgrip strength, and usual gait speed are recommended. However, it is difficult to evaluate these measurements in all patients with IPF in daily practice. In the present study, the serum Cr/CysC ratio correlated well with the recommended measurements: ASMI via BIA (r = 0.561, *P* < 0.001), handgrip strength (r = 0.604, *P* < 0.001), and usual gait speed (r = 0.41, *P* < 0.01). Similar results were reported in patients with chronic obstructive pulmonary disease (COPD), in which the serum Cr/CysC ratio correlated well with ASMI via BIA (r = 0.44, *P* < 0.001) and handgrip strength (r = 0.54, *P* < 0.001) [16]. These results are consistent with the finding that the serum Cr/CysC ratio may be a good predictor of sarcopenia.

One of the strengths of the present study is that the serum Cr/CysC ratio was demonstrated to be a marker of poorer PRO scores as evaluated by mMRC, CAT, and K-BILD on multiple linear regression analysis. The mMRC, CAT, and K-BILD scores are indicators of health-related quality of life in patients with IPF [23, 25–27]. PROs may be helpful in determining the patients’ subjective understanding of IPF and the disease burden on various aspects of their lives. The serum Cr/CysC ratio could contribute to the management of IPF by identifying poorer PROs, which may be associated with complicated sarcopenia.

In contrast to serum Cr/CysC ratio, pulmonary function parameters such as %FVC and %DLco were discarded in the multiple linear regression analysis of PRO scores. A possible explanation for this is the low proportion of patients with severe IPF in the present study. Furthermore, age and exercise tolerance capacity may affect PRO scores more than pulmonary function parameters in IPF patients. However, many reports indicate that a lower muscle mass is associated with impaired pulmonary function in patients with IPF [3, 8, 9].

Elevated serum cystatin C is a sensitive indicator of various chronic inflammatory diseases and has been reported to be associated with the exacerbation of COPD [29]. In the present study, ROC analysis showed that the serum cystatin C level had a moderate AUC of 0.75. In patients with COPD, the serum cystatin C levels were reported to be higher in the sarcopenia group than that in the robust group; however, the serum Cr/CysC ratio was significantly more useful than the serum cystatin C level [16]. Similarly, in the present study, ROC analysis showed that the AUC of the serum Cr/CysC ratio was significantly larger than that of cystatin C (*P* < 0.001).

Cystatin C can be affected by conditions such as renal disease, diabetes mellitus, heart disease and thyroid disease. The data presented in Table 3 showed there is no significant difference in comorbidities between those with low Cr/CysC and those with high Cr/CysC. The possible reason included the small sample size included in the present study.

The present study showed that patients with lower Cr/CysC ratio were older, had a lower BMI, and weaker grip strength. The pulmonary function parameters were not significantly different between the low and high Cr/CysC groups. There was no difference in the level of oxygen desaturation during 6MWT between these groups. However, the PaO_2_ mmHg values were statistically significant between the groups. Possible reasons for no difference being observed in pulmonary function parameters between the high and low Cr/CysC groups could be the small sample size and the absence of patients with severe IPF. Many reports indicate that a lower muscle mass is associated with impaired pulmonary function in patients with IPF [7–10].

The present study had several limitations. First, the results were obtained by analyzing only Japanese patients from a single center with a small sample size. Ninety percent of the patients were men. In addition, we could not examine the validation cohort. Thus, future studies are necessary to examine the serum Cr/CysC cutoff values in more facilities, in other races, in females, and in larger samples. Second, IPF patients with advanced disease (GAP stage III) were not included in this study. Further studies including patients with severe IPF are necessary. Third, this was a cross-sectional study. A longitudinal survey will clarify the clinical significance of an increase or decrease in the serum Cr/CysC ratio. Fourth, we were unable to examine the densitometry in this study. Further study may be needed to clarify the correlation between the serum Cr/CysC ratio and densitometry. Fifth, we used BIA instead of dual-energy X-ray absorptiometry to assess ASMI. However, the AWGS 2019 criteria do recommend BIA as an alternative option for muscle measurement.

## Conclusions

In conclusion, the present study showed that the serum Cr/CysC ratio may be a surrogate marker of sarcopenia in patients with IPF. Moreover, it is important to pay attention to the serum Cr/CysC ratio because a lower serum Cr/CysC ratio is associated with worse PRO scores. Further studies are required to validate these observations to determine whether the Cr/CysC ratio can be used to detect sarcopenia in patients with IPF.

## Data Availability

The datasets generated and/or analyzed during the current study are not publicly available due to no approval by the ethics review board of Nagoya City University Hospital (approval number 60-20-0190) but are available from the corresponding author on reasonable request.

## Abbreviations

IPF: Idiopathic pulmonary fibrosis
AWGS: Asian Working Group for Sarcopenia
PROs: Patient-reported outcomes
ASMI: Appendicular skeletal muscle index
Cr/CysC ratio: Creatinine/cystatin C ratio
6MWT: 6-Minute walk test
BIA: Bioimpedance analysis
DLco: Diffusion capacity for carbon monoxide
%DLco: Percentage of predicted diffusion capacity for carbon monoxide
FVC: Forced vital capacity
%FVC: Percentage of predicted forced vital capacity
FEV_1_: Forced expiratory volume in 1.0 s
GAP: Gender, age, and physiology
SARC-F: Strength, assistance in walking, rising from a chair, climbing stairs, and falls
mMRC: Modified Medical Research Council
CAT: Chronic obstructive pulmonary disease assessment test
K-BILD: King’s Brief Interstitial Lung Disease
ROC: Receiver operating characteristic
VIF: Variance inflation factor
AUC: Area under the curve
COPD: Chronic obstructive pulmonary disease
